# Report of the 4th Asian Conference on Environmental Mutagens at CSIR-Indian Institute of Chemical Biology, Kolkata on December 10–12, 2014

**DOI:** 10.1186/s41021-015-0016-6

**Published:** 2015-09-01

**Authors:** Ashok K. Giri, Takashi Yagi

**Affiliations:** Molecular and Human Genetics Division, CSIR-Indian Institute of Chemical Biology, 4, Raja S. C. Mullick Road, Jadavpur, Kolkata, 700-032 India; Laboratory of Molecular and Cellular Genetics, Department of Biology, Graduate School of Science, Osaka Prefecture University, 1-2 Gakuen-cho, Naka-ku, Sakai, Osaka 599-8570 Japan

**Keywords:** AAEMS, Asian Association of Environmental Mutagen Societies, 4th ACEM, India

## Abstract

The 4th Asian Conference on Environmental Mutagens was held at CSIR Indian Institute of Chemical Biology, Kolkata, during December 10–12, 2014, in collaboration with All India Congress of Cytology and Genetics. Seventy international delegates from Australia, Austria, Belgium, Canada, China, Germany, Iran, Japan, Kazakhstan, Korea and USA along with 150 Indian delegates and students participated in the conference. The conference was inaugurated by Dr. Takehiko Nohmi, President of the International Association of Environmental Mutagenesis and Genomics Societies and Dr. Ashok K. Giri, President of the Asian Association of Environmental Mutagen Societies. The main objective of the conference was to discuss the present scenario of various hazardous chemicals and environmental pollutants in various areas, and their impacts on human health and scientific measure to combat such environmental threats. The organizer truly thanks those who took part in the conference and made the meet a grand success.

## Inaugural session of the 4th ACEM at CSIR-IICB, Kolkata

**Fig. 1:**
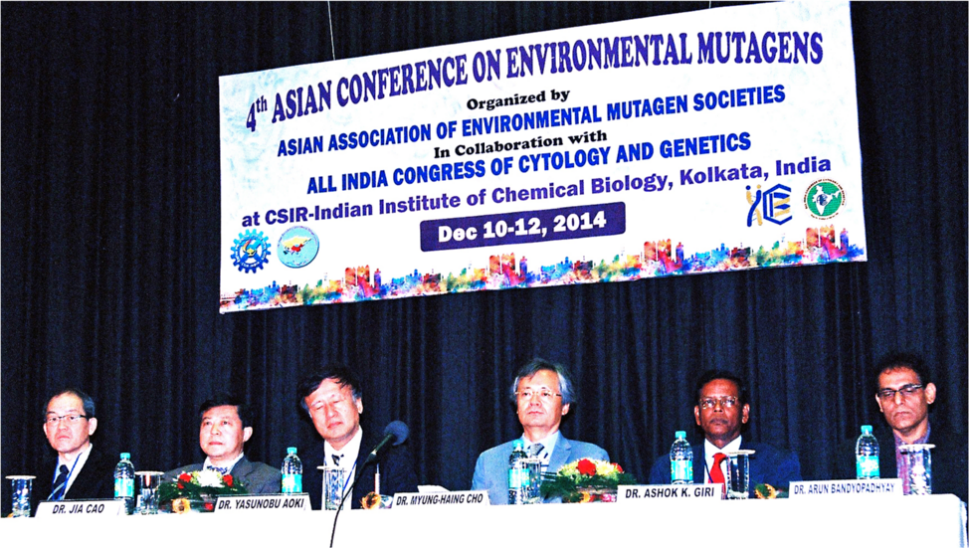
The panel of ACEM Executive Committee Members and Organizing Committee Members at the inaugural function of the 4th ACEM, Kolkata, on December 10, 2014. (*from left*): Dr. T. Nohmi, President of IAEMGS; Dr. J. Cao, Vice President of the Chinese Environmental Mutagen Society; Dr. Y. Aoki, President of the Japanese Environmental Mutagen Society; Dr. M. H. Cho, President of the Korean Environmental Mutagen Society, Dr. Ashok K. Giri, President of AAEMS, and Dr. A. Bandyopadhyay, Senior Principal Scientist, Indian Institute of Chemical Biology

The 4th Asian Conference on Environmental Mutagens (ACEM) was held at CSIR Indian Institute of Chemical Biology (IICB), Kolkata, during December 10–12, 2014, in collaboration with All India Congress of Cytology and Genetics (AICCG). The inaugural session was attended by 70 international delegates from Australia, Austria, Belgium, Canada, China, Germany, Iran, Japan, Kazakhstan, Korea and USA along with 150 Indian delegates and students, who participated in this 3 day international conference. The conference was inaugurated by Dr. Takehiko Nohmi, president of International Association of Environmental Mutagenesis and Genomics Societies (IAEMGS) along with Dr. Ashok K. Giri, president of the Asian Association of Environmental Mutagen Societies (AAEMS) (Fig. 1).

Dr. Giri in his inaugural address gave a brief introduction about the activities and significance of the various environmental mutagen societies in Asia as well as rest of the world. He mentioned that from developing nations to the developed nations, they face some of the most intense environmental issues which culminate into a global despair, and outlined the present scenario of the effects of environmental mutagens like radiation, hazardous chemicals and heavy metals that would be highlighted and discussed as the main objectives in this meeting. He stated that Asia harbors some of the fastest developing countries of the planet, which has earned us the dubious reputation of being the most polluted area. He then focused on another major long standing concern that is the prevalence of wide-spread arsenic toxicity in South East Asia where 160 million individuals are chronically exposed to this Group I carcinogen through contaminated ground-water primarily in Bangladesh, India, Taiwan and China, and new reports on this toxicant still comes out from other countries and newer areas. He reminded that in the year 2001 there were only 21 countries where arsenic contamination was the problem through drinking water and other sources, but in the year 2014 about 70 countries has been included in the list. So this problem is now no more the problem of Asia but a global issue.

He also highlighted that his group has shown a link between rice containing high levels of arsenic and chromosomal damage in humans. His research team found that people of rural West Bengal eating rice as a staple with greater than 200 μg/kg arsenic showed higher frequencies of chromosomal damage. This report has been published in Scientific Reports (Sci. Rep., 3, 2195, 2013, doi:10.1038/srep02195) and based on this report US FDA has started a project to find out the minimum threshold dose of arsenic in rice. Other issues he addressed were fluoride toxicity through contaminated groundwater, air pollution and so on. Dr. Giri continues “*though, I have focused on specific problems so far, the challenges our continent, and indeed, the entire world are facing from environmental contaminants are innumerable. Each of them comes with its own set of unique properties, target populations and ecological effects and to make matters worse, can often act synergistically, thereby increasing their potential to damage.*” He also emphasized that the main objective of the meet was to discuss: about the present scenario of various hazardous chemicals and environmental pollutants in various geographic locations; and about their impacts on direct human health and scientific measure to combat such environmental threats. At the end of his welcome note Dr. Giri requested all the scientists from the globe to come forward and play a leading role in understanding the mechanisms of such environmental toxicant exposure caused maladies and to devise ways to detect them early and to find palliative measures. And with these words, he finally thanked everyone present there for bestowing on him this prestigious opportunity of hosting this conference.

The 3 day conference was divided into relevant sections with a rich plethora of speakers to throw light on the different aspects of the problems people are facing today. Other renowned scientist of various fields demonstrated their experimental findings and probable protective measures. Dr. Takehiko Nohmi, President of IAEMGS, talked about the roles of translesion DNA synthesis in the threshold for genotoxic chemicals. Dr. Yasunobu Aoki, President of the Japanese Environmental Society, proposed transgenic animal models as a tool for risk assessment of environmental mutagens. Dr. Myung-Haing Cho, President of the Korean Environmental Mutagen Societies, talked about effects of intratracheal exposure to multi-walled carbon nanotubes on mice. Dr. Jia Cao, Vice President of the Chinese Environmental Mutagen Society talked about environmental and socio-psycho-behavioral factors associated with male semen quality in China. Other scientists presented their research findings and proposed necessary measures. Scientists from other national institutes and different Indian universities also presented their works and actively participated in the discussion on this particular problem. Thirty one students around the world were supported with travel award to participate in the conference. More than 70 national and international students, post-docs, academicians and professors presented their research work in the first and second day poster session. The final session ended with a feedback from various participants and discussion on action plan to meet the targets. In the valedictory session, the joint organizing secretary, Dr. Arun Bandyopadhyay from CSIR-IICB, in his vote of thanks, expressed his appreciation for the presence of distinguished gathering on the occasion and thanked the delegates individually and collectively, and expressed gratitude of the center to all the sponsors.

## The conference proceedings

The 3 day conference focused on various issues pertaining to the global concern on environmental mutagens. Three sessions encompassing nine plenary talks were arranged, one each day that had nine international acclaimed speakers explaining the various nodes of environmental mutagens. Dr. Michael Fenech elaborated on the need to understand DNA damage and its impact on brain ageing and nutritional preventive measures, while Dr. Sugimura and Dr. Nohmi came across with interesting talks on DNA adductomics and genotoxicity. The second day proceedings were opened with the plenary talk by Dr. Honma, where he elaborated on the need to trace the site-specific DNA adduct formation within the human genome. This was followed by the talks on nano-toxicity and arsenic toxicity by Dr. Cho and Dr. Giri, respectively. A packed auditorium at CSIR-IICB, housing over 200 audiences participated actively in the discussion sessions, asking questions to the speakers based on their research expertise. This was continued in the third plenary session where Dr. Cao, Dr. Aoki and Dr. Tong brought forward interesting aspects on environmental mutagen research, presenting excellent lectures on semen quality, transgenic animal models and mitochondrial toxicity, respectively.

Sixty invited lectures were also organized in two parallel sessions throughout the conference period. The conference emphasized greatly on the need to understand the mechanism of environmental mutagens with various patho-physiological outcomes and carcinogenesis. Special sessions dedicated towards nanotoxicology, radiation-induced toxicity and applied OMICS in toxicology were organized. Arsenic being a major problem in the area of the host institute, special session on Arsenic toxicity was also arranged. Apart from such specialized sessions, aspects on molecular carcinogenesis, epigenetics, DNA damage and repair, etc. were also part of the conference. Each session had an equivocal distribution of Indian and International speakers, which created a good platform to exchange their ideas. The milieu further emphasized on the concerns faced by global community, likewise bringing out pathways to achieve a better route towards amelioration and prevention. One of the main themes of this conference was to harbor a platform for exchange of knowledge and techniques. This was surely reflected from the feedbacks received by the organizing members from the participants.

To provide a platform for the young researchers, the organizing committee of 4th ACEM arranged a short paper session, where young scientists presented their research work. This was well accepted by the scientific community present at the meet, where they came to interact with the minds of the future. The presenters included young researchers from Australia, Canada, Iran and India, who presented their findings on various aspects of environmental mutagens and toxic hazards.

The poster session organized at the 4th ACEM allowed 72 young researchers and graduate students present their works in the form of posters that were evaluated by a panel of eminent scientists. Among the 72 young researchers, 6 were from China, Iran, Kazakhstan, and Korea. It was an excellent platform of interaction between the students as well as the peers, present in the conference.

## Conclusions

The 4th Asian Conference on Environmental Mutagens upheld the rich commune of scientific acumen both from India as well as from International arena. The organizers thank the participating scientists, presidents of the various environmental mutagen societies in Asian countries and the students who took part in this conference and made the meet a grand success. As a President of the AAEMS, once again Dr. Giri thanks all the international and Indian delegates and students to come all the way from different parts of the world to make the conference a grand success.

